# Diapause in Populations of *Tetranychus urticae* (Acari: Tetranychidae) from the Tropical Climate Zone: Morphological, Behavioral, and Metabolomic Characterization

**DOI:** 10.3390/insects17101073

**Published:** 2026-10-16

**Authors:** Júlia Jantsch Ferla, Mariane Possignolo Gomes, Francisco Wesller Batista da Silva, Thaís Regiani Cataldi, Carlos Alberto Labate, Raphael de Campos Castilho, Fernando Luis Cônsoli

**Affiliations:** 1 Department of Entomology and Acarology, Escola Superior de Agricultura “Luiz de Queiroz” (ESALQ), Universidade de São Paulo (USP), Piracicaba 13418-900, São Paulo, Brazil; mariane.gomes@usp.br (M.P.G.); wesller@usp.br (F.W.B.d.S.); raphael.castilho@usp.br (R.d.C.C.); fconsoli@usp.br (F.L.C.); 2 Department of Genetics, Escola Superior de Agricultura “Luiz de Queiroz” (ESALQ), Universidade de São Paulo (USP), Piracicaba 13418-900, São Paulo, Brazil; thais.cataldi@usp.br (T.R.C.); calabate@usp.br (C.A.L.)

**Keywords:** two-spotted spider mites, reproductive arrest, metabolites, phenotypic plasticity, morphological analysis

## Abstract

Diapause is a pre-programmed adaptive strategy that enables many arthropods to survive unfavorable conditions and is well documented in *Tetranychus urticae* from temperate regions. However, phenotypes resembling diapause have recently been reported in tropical regions of Brazil, where the classical environmental cues associated with diapause induction, particularly short days and low temperatures, were not consistently observed across the sampled regions. This study investigated whether these phenotypes represent true diapause by evaluating morphological and reproductive traits in *T*. *urticae* populations from five Brazilian states. In addition, comparative GC-MS metabolomics was performed on diapausing and non-diapausing phenotypes. Reddish-orange body coloration, loss of the dorsal striae lobes, and reproductive arrest confirmed the diapause phenotype in four of the five populations sampled. Metabolomics analysis identified 73 metabolites, including 53 shared between phenotypes, three detected exclusively in diapausing females, and 17 detected exclusively in non-diapausing females. Twelve shared metabolites differed significantly in abundance between phenotypes. Key biomarkers (VIP score > 1.5) included 1-deoxypentitol and glucaric acid, both detected exclusively in diapausing females. These findings provide the first evidence of diapause in *T*. *urticae* populations from tropical regions and reveal metabolic adjustments associated with this physiological state.

## Simple Summary

The two-spotted spider mite, *Tetranychus urticae*, is a major agricultural pest that typically enters diapause, a state of arrested development that enables survival under harsh winter conditions in temperate climates. Surprisingly, *T*. *urticae* females exhibiting diapause-related traits have recently been found on crops in the tropics of Brazil, where the classical environmental cues that induce diapause in this species are absent. This study investigated whether these mites from tropical regions undergo true diapause and characterized their metabolic profiles to gain insight into the physiological mechanisms underlying this adaptation. By examining *T*. *urticae* populations from five Brazilian states, we confirmed that populations from four regions exhibited typical diapause characteristics, including reddish-orange body coloration, loss of the dorsal striae lobes, and cessation of oviposition. Metabolic profiling revealed clear differences between diapausing and non-diapausing mites, indicating metabolic adjustments associated with energy conservation and stress tolerance in diapausing females. These findings provide the first evidence that *T*. *urticae* can enter true diapause under tropical conditions. Understanding diapause in tropical regions may improve the timing of pest monitoring and enhance the effectiveness of management strategies in tropical cropping systems.

## 1. Introduction

Diapause is a developmental arrest associated with alterations in metabolic activity, reduced physiological processes, and increased tolerance to environmental stress [1,2]. This pre-programmed adaptive strategy enables many arthropod species to survive unfavorable environmental conditions while synchronizing their life cycles with periods that are favorable for feeding, development, and reproduction [3,4,5]. Among arthropods, diapause can occur at any developmental stage and varies considerably among species [6,7].

The two-spotted spider mite, *Tetranychus urticae* Koch (Tetranychidae), is a cosmopolitan pest that exhibits a facultative reproductive diapause exclusively in adult females, particularly in populations from temperate regions. Reproductive diapause is induced when immature stages perceive specific environmental cues, primarily short-day photoperiods and low temperatures, which prepare females to survive harsh winter conditions [8].

Morphological changes are associated with diapause in *T*. *urticae*, including the loss of lobes in the integumentary striae [9] and a change in body coloration from greenish to reddish-orange resulting from carotenoid accumulation [10]. Ovarian development is arrested, temporarily preventing egg production [11,12]. In addition, diapausing females cease feeding and undergo several biochemical changes, including alterations in gene expression patterns associated with Ca^2+^ signaling, which plays a key role in various cellular processes and diapause regulation [13]. Metabolic adjustments are also observed, characterized by pronounced metabolic depression and the biosynthesis of compounds associated with cold hardening, including the accumulation of sugar alcohols (polyols) and sugars (trehalose), which enhance cryoprotective capacity and contribute to energy reserves [2,14,15].

In Brazil, *T*. *urticae* is a major pest of *Carica papaya* L. (papaya) and commonly damages older leaves [16,17]. In 2021, populations of *T*. *urticae* comprising a high proportion of individuals exhibiting the typical diapause phenotype (reddish-orange coloration) were reported on papaya plants in the states of Bahia and Espírito Santo [18]. In 2023–2024, growers in the Brazilian states of São Paulo, Minas Gerais, and Mato Grosso also reported *T*. *urticae* individuals exhibiting the diapause phenotype on jack bean (*Canavalia ensiformis* (L.) DC.), raspberry (*Rubus idaeus* L.), and cotton (*Gossypium hirsutum* L.), respectively (authors’ field observations).

These findings are unusual because the environmental conditions generally associated with diapause induction are typically absent in tropical regions, including the Brazilian states where these *T*. *urticae* populations were observed. Furthermore, before entering diapause, the mites appeared to exhibit highly gregarious behavior on the youngest leaves, producing large amounts of webbing. Their feeding resulted in pronounced discoloration, impaired normal leaf development, and, in some cases, leaf drop [18]. Mite management becomes more challenging when individuals enter diapause, as diapausing mites typically seek shelter under younger leaves and reduce or cease feeding activity, thereby decreasing their exposure to miticides.

Because climatic conditions in these regions do not match those reported in the literature as necessary for diapause induction in *T*. *urticae*, it is important to determine whether these mites are indeed undergoing diapause to better understand its potential consequences for agricultural production and to develop appropriate management strategies. Metabolomics is a powerful approach for elucidating the physiological state of these *T*. *urticae* populations from tropical regions. Although morphological and behavioral observations can provide initial evidence of diapause, metabolomic profiling offers a comprehensive assessment of the biochemical reprogramming associated with this physiological state [19,20].

Therefore, this study aimed to determine whether the observed phenotypes represent true diapause and whether the diapause phenotype in *T. urticae* occurs widely across different crops and regions in Brazil or is restricted to a specific population. Furthermore, we conducted a comparative metabolomic analysis of diapausing (DP) and non-diapausing (NDP) phenotypes using the Espírito Santo population as a model.

## 2. Materials and Methods

### 2.1. Mite Collection and Colony Establishment

Mite populations were sampled based on field reports of *T*. *urticae* displaying the reddish-orange, diapause-like phenotype on various crops across different tropical regions of Brazil (Espírito Santo, São Paulo, Minas Gerais, and Mato Grosso). In addition, a population was sampled in the state of Rio Grande do Sul, a subtropical region characterized by winter environmental conditions, including lower temperatures and shorter photoperiods, that are compatible with the environmental cues known to induce diapause in *T*. *urticae* in temperate regions. This population was included to investigate whether the diapause phenotype also occurs under these conditions in Brazil.

**Figure 1:**
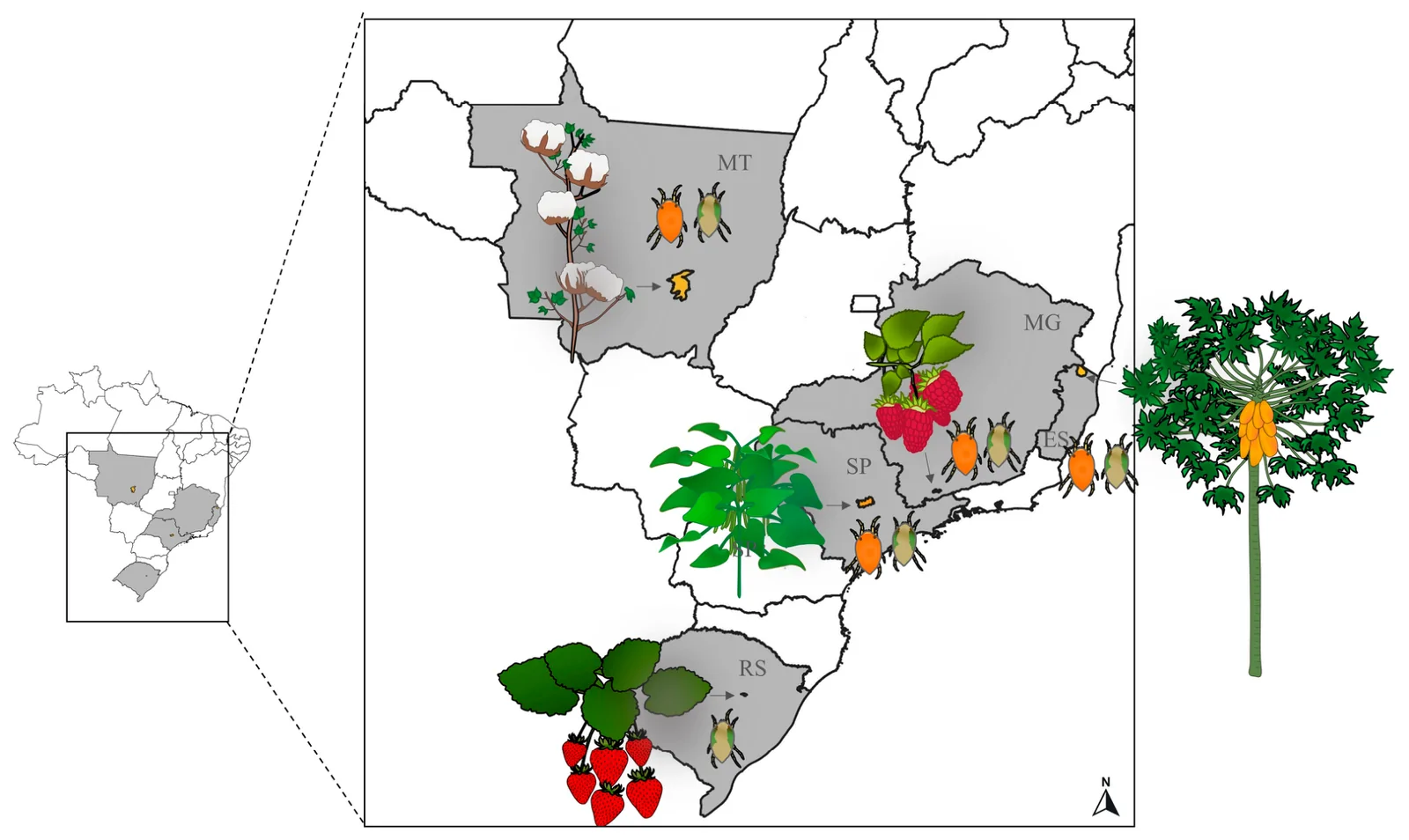
Spatial distribution of the occurrence of diapause in *Tetranychus urticae* across Brazilian states and crops. Gray shading indicates the states where mites were collected (MT, SP, MG, ES, and RS), whereas yellow shading indicates the specific sampling localities within these states. Orange mite icons indicate localities where diapausing individuals were found, while green mite icons indicate localities where non-diapausing individuals were found.

The selected sampling sites (Figure 1) and the corresponding climatic data, obtained from the National Institute of Meteorology (INMET), are provided below:

- Montanha, Espírito Santo state (ES): 18°07′21″ S, 40°21′57 W; altitude: 180 m a.s.l.; crop: *C*. *papaya* (papaya); collection date: 17 July 2023; climatic conditions in July 2023: mean maximum temperature 27.2 °C, mean minimum temperature 18.3 °C, overall mean temperature 21.8 °C; photoperiod: 11:13 h light:dark; meteorological station: 19°21′36″ S, 40°04′12″ W.
- Piracicaba, São Paulo state (SP): 22°42′14.3″ S, 47°38′01 W; altitude: 554 m a.s.l.; crop: *C. ensiformis* (jack bean); collection date: 20 March 2023; climatic conditions in March 2023: mean maximum temperature 30.8 °C, mean minimum temperature 19.5 °C, overall mean temperature 24 °C, photoperiod: 12:12 h light:dark; meteorological station: 22°42′00″ S, 47°37′12″ W.
- Maria da Fé, Minas Gerais state (MG): 22°18′29″ S, 45°22′25″ W; altitude: 1258 m a.s.l.; crop: *R. idaeus* (raspberry); collection date: 15 July 2024; climatic conditions in July 2024: mean maximum temperature 22.6 °C, mean minimum temperature 7.5 °C, overall mean temperature 13.5 °C; photoperiod: 11:13 h light: dark; meteorological station: 22°18′36″ S, 45°22′12″ W.
- Primavera do Leste, Mato Grosso state (MT): 15°32′40.89″ S, 54°19′7.85″ W; altitude: 636 m a.s.l.; crop: *G. hirsutum* (cotton); collection date: 1 August 2024; climatic conditions in August 2024: mean maximum temperature 32.3 °C, mean minimum temperature 18.5 °C, overall mean temperature 25 °C; photoperiod—12:12 h light: dark; meteorological station: 15°33′36.0″ S, 55°10′48.0″ W.
- Lajeado, Rio Grande do Sul state (RS): 29°26′51.90″ S, 51°56′52.20″ W; altitude: 48 m a.s.l.; crop: *Fragaria* sp. (strawberry); collection date: 20 July 2023; climatic conditions in July 2023: mean maximum temperature 20.8 °C, mean minimum temperature 12.1 °C, overall mean temperature 15.4 °C; photoperiod: 10:14 h light: dark; meteorological station: 30°03′00.00″ S, 51°10′12.00″ W.

At each site, leaf samples were collected, placed in tightly sealed plastic bags, and transported in Styrofoam boxes to a restricted-access laboratory at the Acarology Laboratory, Escola Superior de Agricultura “Luiz de Queiroz” (ESALQ), Universidade de São Paulo (USP), Piracicaba, São Paulo. For the ES population, a stock colony was established and maintained at 25 ± 2 °C, 70 ± 10% RH, and a 12:12 h light:dark photoperiod on jack bean plantlets within mite-proof screened cages. Specimens displaying the diapause phenotype continued to be produced at varying frequencies regardless of the season.

### 2.2. Morphological Characteristics

From each population, five diapausing adult females, five non-diapausing adult females, and five males were mounted on glass slides in Hoyer’s medium [21] and placed in a drying oven at 50–60 °C for approximately 10 days. The slides were then examined under phase-contrast microscopy to confirm species identity based on morphological characteristics [22] and to verify the presence of lobes on the integumentary striae.

### 2.3. Oviposition Assays

#### 2.3.1. Group Evaluation (MT, SP, MG, and ES Populations)

To evaluate the reproductive activity of field-collected females exhibiting the diapause phenotype, oviposition was assessed. The experimental unit consisted of a jack bean leaflet placed abaxial side up on a layer of moistened cotton wool inside a plastic box (26.2 × 17.7 × 8.5 cm). The leaflet margins were secured with wet cotton strips to prevent leaf desiccation and mite escape, and moisture was maintained by adding distilled water daily.

For the group evaluation, 20 randomly selected diapausing females from each field-collected population were placed on a single experimental unit, with each population evaluated separately. As a control, the same procedure was followed using 20 non-diapausing females from each field-collected population. All experimental units were maintained at 25 ± 2 °C under a 12:12 h light:dark photoperiod.

The females were monitored daily for up to 14 days to verify oviposition. Although oviposition was monitored daily, the time required to resume oviposition and regain the typical green coloration was not systematically quantified and was therefore not compared among populations.

#### 2.3.2. Individual Evaluation (ES Population)

Due to the absence of oviposition observed during the group evaluation, an individual evaluation was subsequently conducted exclusively for the ES population, in which the diapause phenotype was continuously expressed in the stock colony. The experimental unit consisted of a jack bean leaf disc (6.3 cm in diameter) placed abaxial side up on a layer of moistened cotton wool inside a Petri dish (1.5 cm high × 9 cm in diameter). The leaf disc margins were secured with wet cotton strips to prevent leaf desiccation and mite escape, and moisture was maintained by adding distilled water daily.

For the individual evaluation, 30 diapausing females were randomly selected and individually isolated on separate experimental units, resulting in 30 replicates (one female per experimental unit). As a control, the same procedure was followed using 30 non-diapausing females from the ES population. All experimental units were maintained at 25 ± 2 °C under a 12:12 h light: dark photoperiod.

The females were monitored daily for up to 14 days. For each replicate, when the first egg was observed, oviposition was confirmed, and the female’s coloration was recorded, at which point the evaluation was terminated. Although females were monitored daily, the time required to resume oviposition and regain the typical green coloration was not systematically quantified and was therefore not compared among populations.

### 2.4. Metabolomics

#### 2.4.1. Sampling, Metabolite Extraction and Derivatization

Given that all populations exhibiting the diapause phenotype shared similar morphological characteristics and oviposition patterns, the metabolic profile of diapausing females was compared with that of non-diapausing females using only the Espírito Santo (ES) population. Both diapausing and non-diapausing females used in the metabolomic analysis were obtained from the ES stock colony after being reared under identical laboratory conditions (25 ± 2 °C, 70 ± 10% RH, and 12:12 h light: dark photoperiod) on jack bean plants. No field-collected females were used directly in this metabolomic analysis.

Each treatment (diapausing and non-diapausing) consisted of five replicates. Each replicate comprised a pool of 100 randomly selected adult females. Females were transferred to methanol-treated plastic vials, immediately snap-frozen in liquid nitrogen, and stored at −80 °C until further processing.

Metabolite extraction was performed according to Hoffman et al. [23], with some modifications, as described below. Each sample was homogenized by mechanical lysis with three 5.0-mm tungsten beads and agitated at 30 Hz for 1 min in a TissueLyser (QIAGEN, Hilden, Germany). The homogenates were extracted with 1 mL of ice-cold 6:2:2 (methanol:chloroform:water) extraction solution, then sonicated at 20 Hz for 15 min in a cold-water bath. Samples were then centrifuged at 16,000× *g* for 10 min at 4 °C, and the supernatants were recovered. For derivatization, 15 μL of methoxyamine hydrochloride was added to each sample, followed by vortexing and incubation in the dark at room temperature for 16 h. Subsequently, 15 μL of N-methyl-N-trimethylsilyl trifluoroacetamide containing 1% trimethylchlorosilane was added, and the samples were allowed to stand for 1 h at room temperature. The samples were then vortexed, and 15 μL of a methyl esterase solution in heptane (15 ng^−1^) was added.

#### 2.4.2. GC-MS and Statistical Analysis

The samples were analyzed using gas chromatography coupled with time-of-flight mass spectrometry (GC/TOF-MS), following Budzinski [24]. To concentrate the metabolites, samples were dried in a vacuum concentrator until a final volume of approximately 1 µL was reached. Retention indices were calculated using a series of n-alkanes (C12–C40). The alkane mixture was analyzed at the beginning and end of each analytical batch to assess chromatographic performance and the consistency of retention times and retention indices.

Aliquots (1 µL) of each sample were injected into an Agilent 7890A gas chromatograph equipped with a CTC Combi Pal Xt Duo autosampler (CTC Analytics AG, Zwingen, Switzerland). The injection port was maintained at 260 °C under splitless conditions, with a septum purge flow of 20 mL min^−1^ initiated after 75 s. Chromatographic separation was achieved using a fused-silica capillary column (30 m length × 0.25 mm i.d.) coated with a 0.25 μm DB-5MS UI stationary phase (J&W Scientific, Folsom, CA, USA). Helium was used as the carrier gas at a flow rate of 1 mL min^−1^. The oven temperature was initially maintained at 70 °C for 2 min, then increased at a rate of 15 °C min^−1^ to 320 °C, followed by a final hold of 4 min.

The column effluent was introduced into a Pegasus HT time-of-flight mass spectrometer (Leco Corporation, St. Joseph, MI, USA). The transfer line and ion source temperatures were maintained at 250 °C and 200 °C, respectively. Ionization was achieved by electron ionization at 70 eV with an ionization current of 2.0 mA. Mass spectra were acquired at 30 spectra s^−1^ for run 1 and 20 spectra s^−1^ for run 2 over a mass range from 50 to 800 *m*/*z*. The acceleration voltage was activated after a solvent delay of 290 s, and the detector voltage was set to 1450 V for run 1 and 1490 V for run 2.

Data were processed using ChromaTOF 4.32 software, which was used for peak detection, baseline correction, and retention index and retention time alignment. Metabolites were identified by comparison with the NIST library, and only those with identification scores ≥ 700 were considered. Metabolite intensities were normalized to the total ion current (TIC) of each sample.

Synthetic metabolites and metabolites detected in blank samples were excluded from the analysis. Metabolites detected in at least 50% of the replicates of at least one treatment were retained, whereas those detected in fewer than 50% of the replicates in both treatments were excluded. Statistical analyses were performed using the MetaboAnalyst 5.0 web server [25] (MetaboAnalyst version 5.0, available at http://www.metaboanalyst.ca/MetaboAnalyst/). Data were log2-transformed and Pareto-scaled. Differences in metabolite abundance between treatments were assessed using one-factor *t*-tests, with *p*-values adjusted for multiple comparisons using the false discovery rate (FDR), with a significance threshold of 0.05. Partial least squares discriminant analysis (PLS-DA) was performed to visualize separation between treatments. Candidate biomarkers were identified based on the variable importance in projection (VIP) scores obtained from the PLS-DA regression model. A heatmap was generated based on the *t*-test results to visualize metabolite abundance and identify relevant patterns between treatments.

The AI tools ChatGPT (OpenAI, GPT-5.5), Gemini (3.1 Pro), and DeepL Write (26.7.2) were used exclusively to provide linguistic support during the preparation of the manuscript, including reviewing grammar and improving text fluency.

## 3. Results

### 3.1. Morphological Characteristics

Females exhibiting the diapause phenotype were detected directly upon field collection in the ES, SP, MG, and MT populations. At all locations, the diapause phenotype was sufficiently conspicuous to have been noticed by growers before sampling, although its prevalence was not systematically quantified. In contrast, the RS population was the only one in which the diapause phenotype was not observed at the time of sampling.

**Figure 2:**
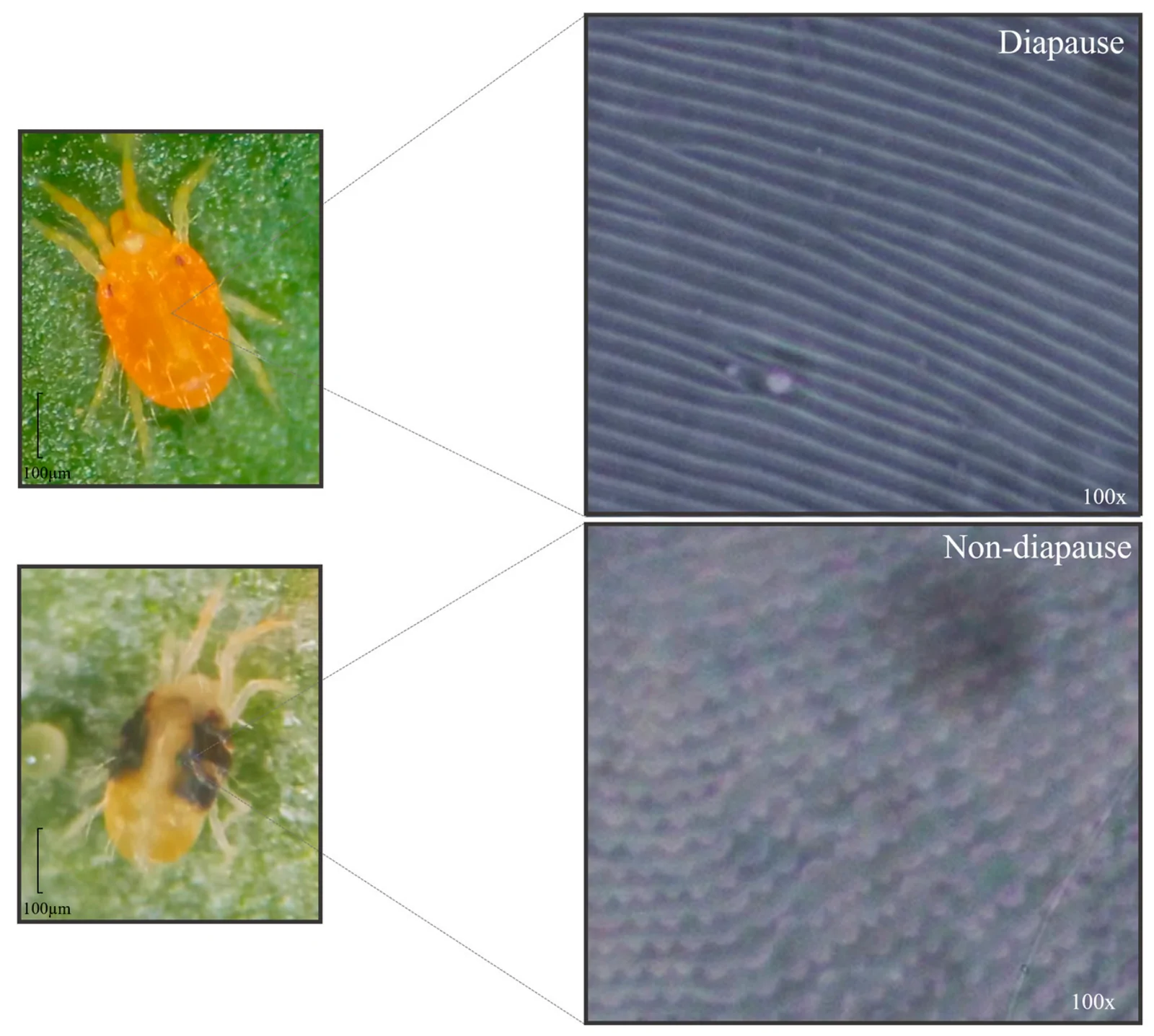
Diapausing and non-diapausing female phenotypes. (**Left**) body coloration. (**Right**) phase-contrast microscopy images of dorsal integumentary striae with (non-diapausing) and without (diapausing) lobes, at 100× magnification.

Diapause individuals were distinguished from non-diapausing individuals mainly by their reddish-orange body coloration and the absence of lobes on the integumentary striae (Figure 2). In contrast, non-diapausing individuals have a greenish body coloration, and their integumentary striae bear lobes.

### 3.2. Oviposition

None of the females exhibiting the diapause phenotype laid eggs during the observation period, whether kept in groups or separately. Daily examination of the arenas confirmed no oviposition until the females changed to the green body coloration typical of non-diapausing individuals. In contrast, all females in the non-diapausing control treatments oviposited during the observation period.

### 3.3. Metabolomics

**Figure 3:**
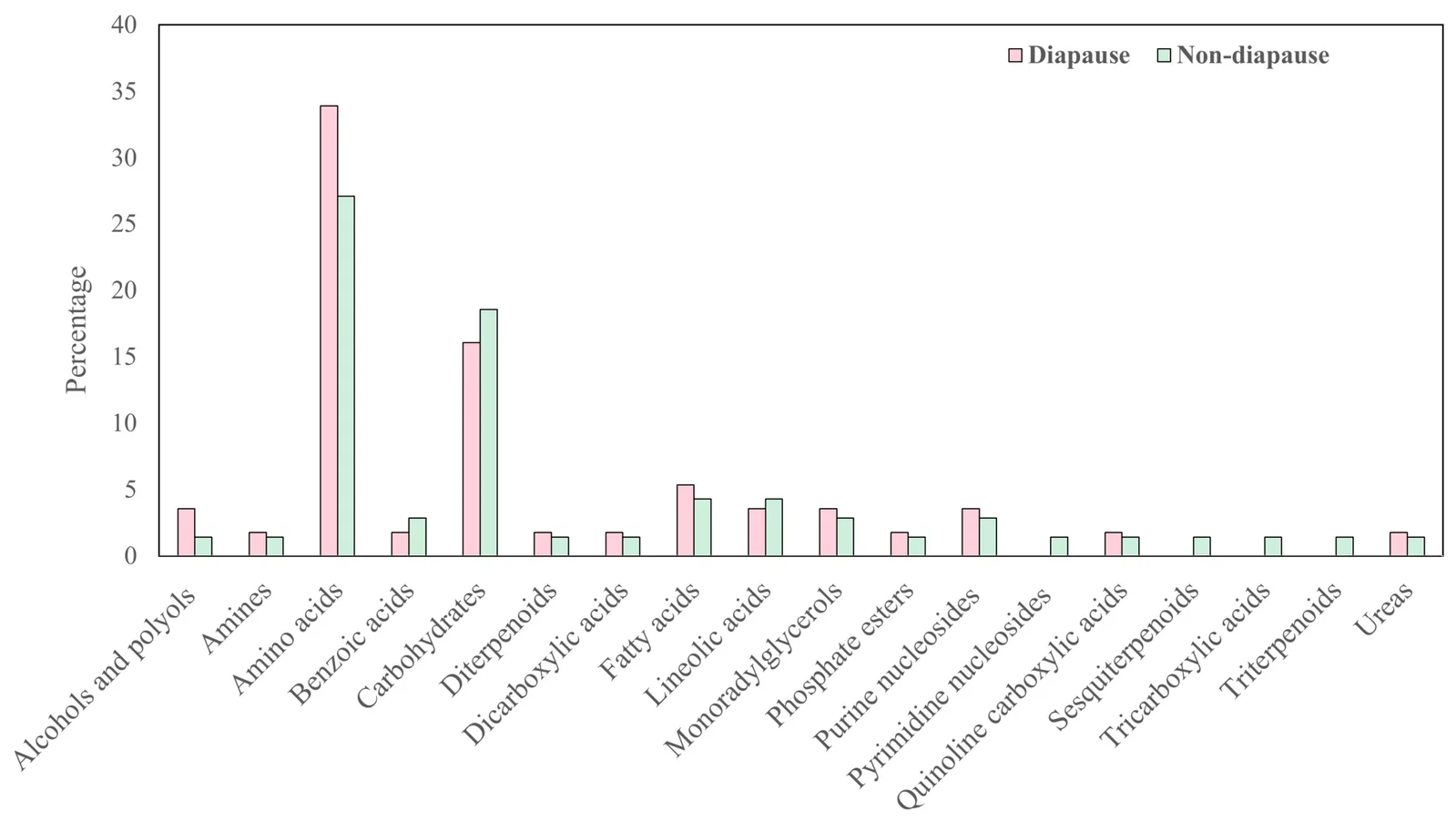
Composition (%) of the main metabolite classes in diapausing (pink) and non-diapausing (green) *Tetranychus urticae* females.

A total of 73 metabolites were identified in diapausing and non-diapausing female mites, of which 53 (72.6%) were detected in both phenotypes. Three metabolites (4.1%) were detected exclusively in diapausing females (1-deoxypentitol, cyclo-ornithine, and glucaric acid), whereas 17 metabolites (23.3%) were detected exclusively in non-diapausing females (D-tyrosine zwitterion, oxine, 2-imidazolidinone, glyceryl linoleate, anthranilic acid, citric acid, galacturonic acid, ferulic acid, galactaric acid, glyceric acid, glycerol 2-O-galactopyranoside, gulose, lysine, rhamnose, neophytadiene, supraene, and uridine) (Supplementary Table S1). Amino acids were the predominant metabolite group in both phenotypes (diapausing = 33.9%; non-diapausing = 27.1%), followed by carbohydrates (diapausing = 16.0%; non-diapausing = 18.5%) and fatty acids (diapausing = 5.3%; non-diapausing = 4.2%) (Figure 3; Supplementary Table S1).

**Figure 4:**
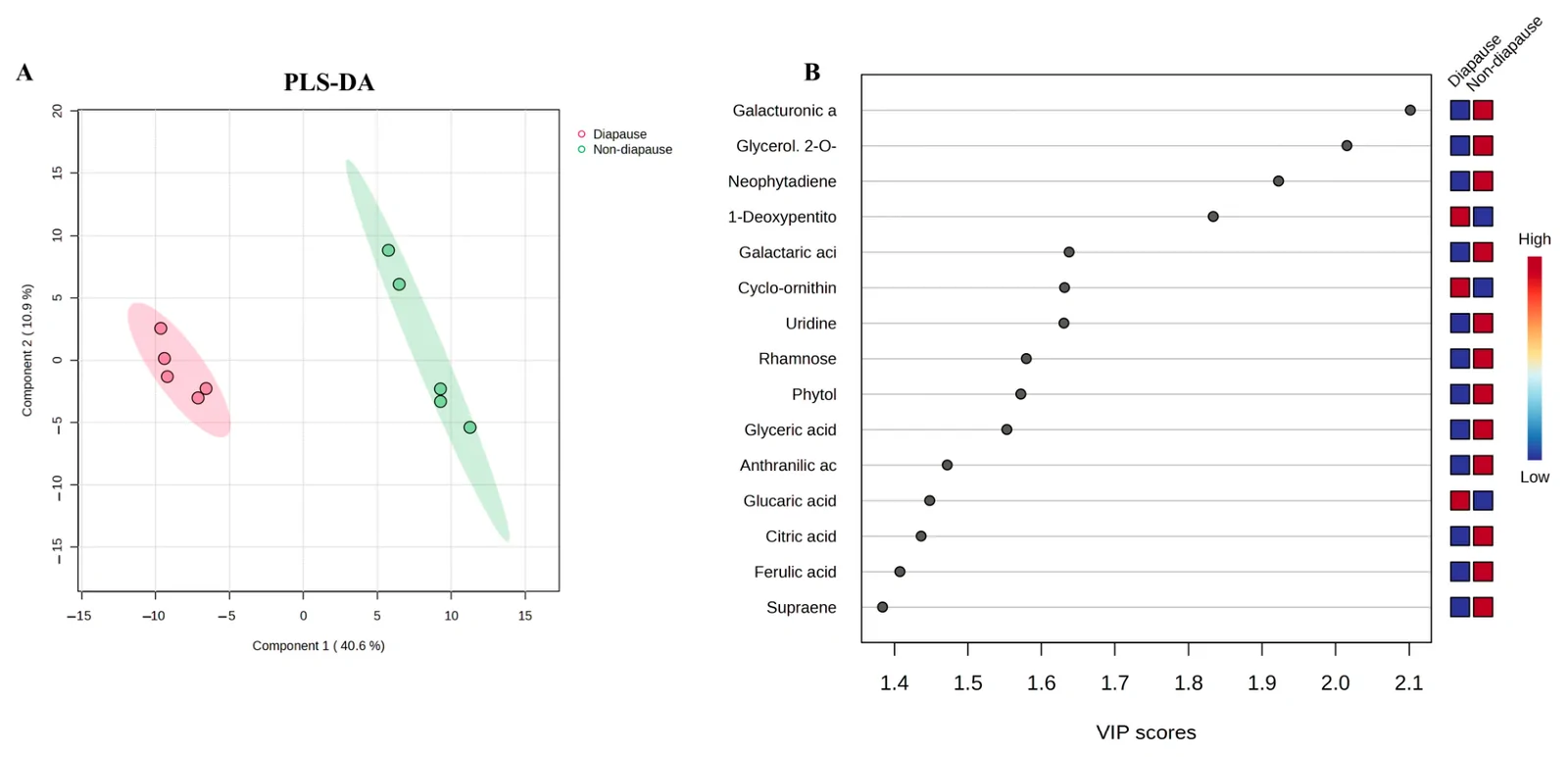
(**A**) Partial least squares discriminant analysis (PLS-DA) score plot showing the separation between diapausing (pink) and non-diapausing (green) samples. (**B**) Variable importance in projection (VIP) scores of the metabolites contributing to the separation between groups in Component 1, with red indicating higher abundance and blue indicating lower abundance.

**Figure 5:**
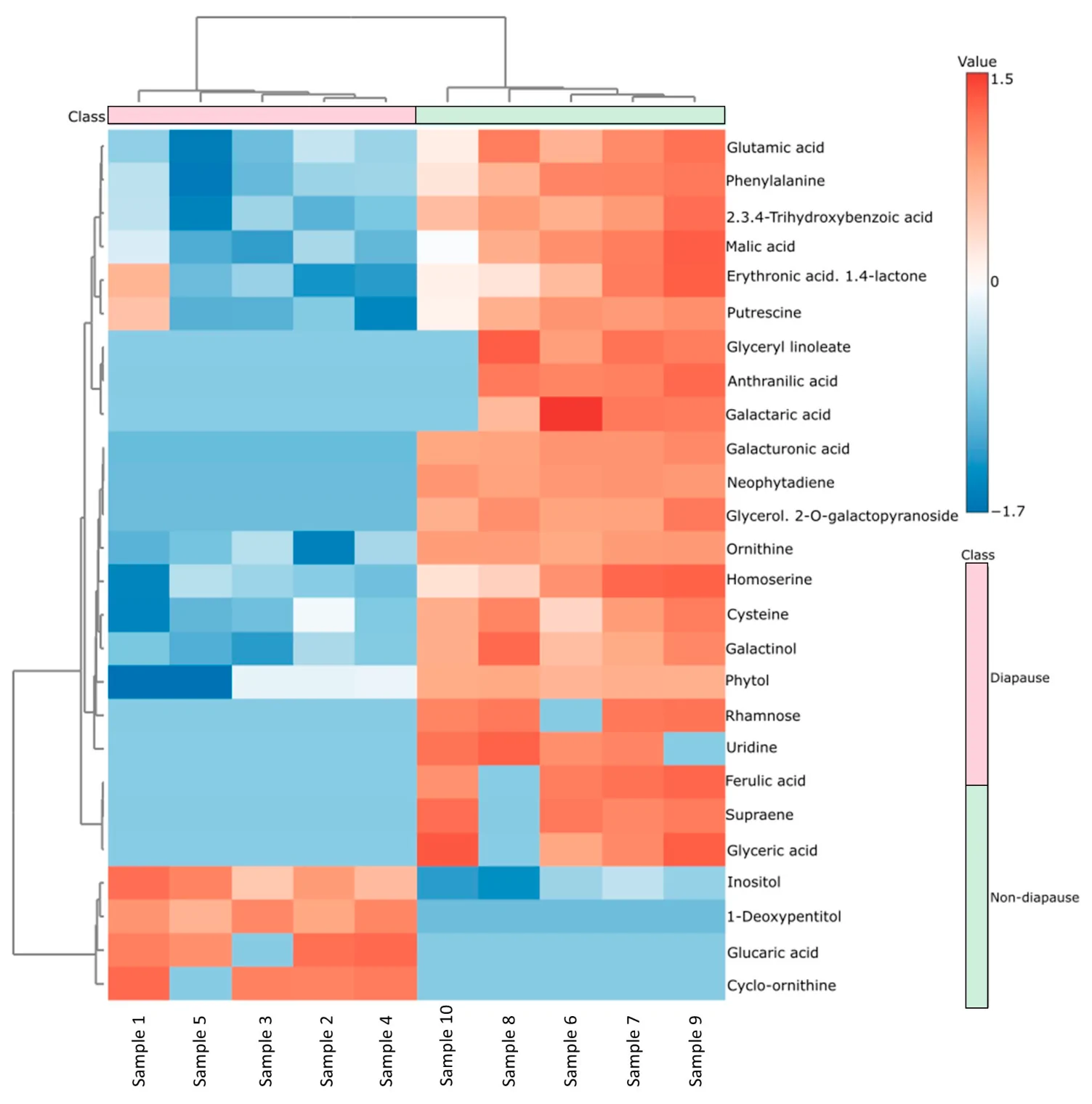
Heatmap showing the relative abundance of metabolites significantly affected by phenotype according to a *t*-test (FDR < 0.05) in diapausing and non-diapausing females. The pink bar represents samples from diapausing females, whereas the green bar represents samples from non-diapausing females. The color scale represents relative metabolite abundance, with red indicating higher abundance and blue indicating lower abundance.

Females displaying the morphological traits of the diapausing phenotype of *T*. *urticae* exhibited a metabolic profile clearly distinct from that of non-diapausing females, as demonstrated by Components 1 and 2 of the PLS-DA (Figure 4A; model validation: R^2^ = 0.99, Q^2^ = 0.88 for two components). Pairwise comparisons of the 53 metabolites shared by both phenotypes showed that eight (15%) differed significantly between the two groups (*t*-test, FDR < 0.05) (Table 1, Figure 5).

The PLS-DA model also identified the metabolites with the highest variable importance in projection (VIP) scores, with 10 metabolites exhibiting VIP scores > 1.5 (Figure 4B). Of these, seven were detected exclusively in non-diapausing females: galacturonic acid, glycerol 2-O-galactopyranoside, neophytadiene, galactaric acid, uridine, rhamnose, and glyceric acid. Phytol was detected in both phenotypes but was more abundant in non-diapausing females. The remaining two metabolites, 1-deoxypentitol and cyclo-ornithine, were detected exclusively in diapausing females (Figure 4B).

## 4. Discussion

The data show that diapause in *T*. *urticae* occurs across different regions and crops in Brazil, indicating that this phenomenon is not restricted to populations from temperate regions. The morphological and biological characteristics observed are consistent with those described in the literature for reproductive diapause in *T*. *urticae*. Diapausing females exhibit a reddish-orange body coloration, integumentary striae without lobes, and an inability to oviposit, with oviposition resuming when the body color changes back to green [9,10,12].

Specific metabolites may serve as biological markers characterizing the diapause state. The *T*. *urticae* of the tropical region examined in this study exhibited 26 significantly altered metabolites between diapausing and non-diapausing females, including compounds detected exclusively in each state. Among these metabolites, several amino acids, polyols, and sugars were also reported in diapausing *T. urticae* from populations in temperate regions [2]. Specifically, 13 compounds were common to the two datasets: six amino acids (alanine, ornithine, phenylalanine, serine, threonine, and valine), four polyols (glycerol, inositol, mannitol, and xylitol), two sugars (glucose and maltose), and one unclassified compound (putrescine).

Food quality and quantity are important factors that can induce diapause in mites [26]. As feeding decreases during diapause, mites reallocate energy to maintain essential physiological functions, resulting in metabolic adjustments that support cellular homeostasis [1,27]. Whereas mites from colder regions accumulate cryoprotective carbohydrates such as inositol, mannitol, and sorbitol [2], diapausing mites occurring in a tropical region exhibited the exclusive production of 1-deoxypentitol and a 2.44-fold higher level of inositol compared with non-diapausing females. These patterns suggest an adjustment in polyol metabolism associated with diapause in a population from the tropics, rather than the pronounced accumulation of cryoprotection compounds associated with cold hardness.

Polyols are synthesized in response to environmental stress and can protect cells from damage by maintaining protein stability and osmotic balance [28]. Although their accumulation in arthropods is commonly associated with cold hardiness and overwintering [29], polyols can also function as chemical chaperones under other stress conditions [30]. Thus, the polyols identified in this study may contribute to cellular protection against the abiotic stresses experienced during diapause in tropical ecosystems.

Several plant-derived compounds, including neophytadiene, galacturonic acid, and oxine, were not detected in diapausing females, suggesting reduced feeding during diapause. Neophytadiene is produced during chlorophyll degradation [31], galacturonic acid is the main component of pectin [32], and oxine has antifungal and antibacterial activity in plants [33,34]. Although oxine is also used as a fungicide, no fungicides were applied to the host plants, which were maintained under standardized controlled conditions. Thus, its exclusive detection in non-diapausing females supports its potential association with plant feeding, although its role in the metabolic differences between phenotypes remains unknown.

Consistent with this pattern, phytol, a chlorophyll-derived metabolite detected in both phenotypes [35], was significantly lower in diapausing females, showing an approximate 21.6-fold reduction relative to non-diapausing females (fold change = 0.046). Although feeding rate was not directly quantified, the absence or marked depletion of these plant-derived metabolites supports reduced food intake during diapause and is consistent with metabolic depression in *T*. *urticae* populations occurring in tropical regions.

Cyclo-ornithine (3-amino-2-piperidone) was detected in diapausing females but not in non-diapausing females. Although its biological function remains poorly understood, its association with ornithine metabolism suggests a potential link to *γ-aminobutyric acid* (GABA) production [36,37]. GABA has been implicated in diapause regulation in several insects, including *Helicoverpa armigera* (Hübner) (Lepidoptera: Noctuidae), *Sarcophaga bullata* (Parker) (Diptera: Sarcophagidae), and *Bombyx mori* (L.) (Lepidoptera: Bombycidae) [38,39], and may also be involved in circadian regulation in *Periplaneta americana* (L.) (Blattodea: Blattidae) [40]. However, the relationship among GABA, circadian regulation, and photoperiodism in arthropods remains poorly understood. Moreover, unlike in some insects in which neuropeptides such as *Diapause Hormone* (DH) regulate diapause [6], no *T. urticae* DH orthologue has been identified to date. Thus, although GABA-related pathways may contribute to diapause regulation, the mechanisms underlying diapause induction, maintenance, and termination in mites remain largely unknown.

In *T*. *urticae*, reddish-orange coloration is associated with carotenoid accumulation. The accumulation of keto-carotenoids such as astaxanthin and 3-hydroxyechinenone is essential for diapause induction, and these compounds are synthesized from β-carotene, a precursor of vitamin A [41,42,43]. Their accumulation is driven by the expression of genes encoding enzymes involved in the carotenoid biosynthetic pathway [44,45]. These pigments are important not only for photoperiod perception but also for protecting mites against oxidative stress and UV radiation [45,46]. Although the intense pigmentation observed in the mites in this study is consistent with the accumulation of these compounds, keto-carotenoids are highly lipophilic and typically require targeted extraction and specific HPLC methods for detection [47,48]. Because the untargeted metabolomic approach used in this study was optimized for primary metabolites, these pigments likely fell outside its analytical range, explaining their absence from the dataset.

In this study, diapausing females were naturally recorded in field populations from tropical regions (ES, SP, and MT), where the climatic conditions classically associated with diapause induction do not typically occur. In the ES stock colonies, diapausing females continued to appear across successive generations despite the change from papaya to jack bean and the absence of the classical environmental cues associated with diapause induction. The MG population, however, was collected in a tropical region under environmental conditions that may have been sufficient to induce diapause in *T*. *urticae* [8], with a mean temperature of 13.5 °C and approximately 11 h of daylight during the observation period. In contrast, diapausing females were not observed in the RS population, which was collected in a subtropical region where winter temperatures and photoperiod conditions were more conducive to diapause induction [8].

The proportion of diapausing versus non-diapausing mites on the plants varied among locations, ranging from approximately 5% on cotton plants in MT to approximately 30% on jack bean plants in SP. A common feature across populations was that diapausing mites consistently migrated from older, more deteriorated leaves to younger leaves and produced more webbing than non-diapausing mites. Similarly, *T*. *urticae* populations collected from different localities and host plants in Japan showed substantial variation in diapause incidence, ranging from nearly 100% in northern populations to 0% in southwestern populations [49].

However, because the temperature and photoperiod thresholds for diapause induction were not systematically tested for each sampled region, the exact environmental limits driving this process in the field cannot be definitively established. Moreover, although diapausing females were observed on different plant species, including jack bean, cotton, papaya, and raspberry, all were commercially cultivated. Therefore, the potential role of crop management practices cannot be ruled out, as shared agricultural stressors might also influence diapause induction in these agroecosystems.

Diapause is a conserved genetic trait [50], and its persistence in populations of *T*. *urticae* from the tropics suggests that the underlying genetic mechanisms remain functional, while the environmental cues triggering its expression may extend beyond the classically recognized factors of temperature and photoperiod. This complexity is further highlighted by the RS population, which did not exhibit the diapause phenotype under the specific conditions and timeframe evaluated in this study. Therefore, significant gaps remain in understanding diapause regulation in the tropics. To determine whether this phenotypic response evolved independently as a local adaptation or represents a remnant trait from temperate mite strains introduced to Brazil, future studies should investigate the genetic diversity and population structure of *T*. *urticae* across different ecozones. Elucidating the specific genetic and environmental mechanisms underlying tropical diapause will clarify its physiological basis and may contribute to the development of effective, region-specific management strategies.

The practical and biological implications of this discovery pose significant challenges for agricultural management. Diapausing mites are more stress-tolerant, feed little or not at all, and seek shelter to avoid ultraviolet radiation [2,13,46]. These traits may reduce their susceptibility to chemical control through complementary mechanisms: Behaviorally, seeking shelter reduces exposure to contact acaricides, whereas reduced feeding may limit exposure to active ingredients that act through ingestion. This emphasizes the need for integrated control strategies [18]. Biologically, the occurrence of diapause under tropical conditions demonstrates the adaptive plasticity of *T*. *urticae* and its capacity to respond to novel ecological challenges in tropical agroecosystems [51].

## Figures and Tables

**Table 1:** Metabolites present in both phenotypes whose abundance was significantly affected by phenotype according to a *t*-test (FDR < 0.05). DP = diapausing females; NDP = non-diapausing females; FDR = false discovery rate.

Metabolites	Fold Change (DP/NDP)	Abundance Pattern	FDR
Galactinol	0.202810765	Elevated in NDP	6.27 × 10^−5^
Ornithine	0.217581347	Elevated in NDP	0.00013
2,3,4-Trihydroxybenzoic acid	0.460665123	Elevated in NDP	0.00031
Inositol	2.444601734	Elevated in DP	0.00048
Cysteine	0.343975015	Elevated in NDP	0.00143
Phenylalanine	0.327759064	Elevated in NDP	0.00143
Homoserine	0.306941346	Elevated in NDP	0.00155
Glutamic acid	0.381223018	Elevated in NDP	0.00161
Malic acid	0.426977684	Elevated in NDP	0.00285
Phytol	0.046246485	Elevated in NDP	0.01411
Putrescine	0.301554763	Elevated in NDP	0.01411
Erythronic acid. 1,4-lactone	0.671163989	Elevated in NDP	0.03738

## Data Availability

The data supporting the conclusions of this article will be made available by the corresponding author upon reasonable request.

## Supplementary Materials

The following supporting information can be downloaded at https://www.mdpi.com/article/10.3390/insects17101073/s1, Table S1: Metabolites detected in the metabolomic analysis of diapausing and non-diapausing females. Presence (1) and absence (0). The putative source of each metabolite is indicated (Me = Mite; P = Plant; Mo = Microbiota).

## Abbreviations

The following abbreviations are used in this manuscript:

NDP	Non-diapausing phenotype
DP	Diapausing phenotype
ES	Espírito Santo
MT	Mato Grosso
SP	São Paulo
MG	Minas Gerais
RS	Rio Grande do Sul
GABA	γ-aminobutyric acid
RDL	Resistance to dieldrin
DH	Diapause Hormone
